# Energy-Efficient Dynamic RTO with Enhanced Stability for CoAP-Based IoT Networks

**DOI:** 10.3390/s26123960

**Published:** 2026-06-22

**Authors:** Suyoung Choi

**Affiliations:** Software Innovation Center, Dong-A University, Busan 49315, Republic of Korea; csy100479@hanmail.net

**Keywords:** adaptive RTO, CoAP, CoCoA+, FASOR, congestion control, Cooja simulator, IoT, Gilbert–Elliott model, dynamic packet loss, wireless sensor networks, retransmission timeout, RTT variability

## Abstract

**Highlights:**

**What are the main findings?**

- A novel dual-adaptive Dynamic *RTO* algorithm is engineered to simultaneously adjust the window inspection cycle (*N*) based on instantaneous *RTT* variance and the historical smoothing weight (α) relative to real-time packet loss trends.
- Comprehensive multi-hop simulations across 1 × 6, 3 × 6, and 5 × 6 topologies under a time-varying Gilbert–Elliott loss model demonstrate that the proposed mechanism reduces total communication time by up to 14.28% and 8.89% compared to state-of-the-art CoCoA+ and FASOR protocols, respectively.
- The proposed algorithm significantly curtails transmission overhead in complex network bottlenecks, restricting the cumulative retransmission footprint to just 59 counts under severe localized impairments, effectively shielding the transport layer from retransmission storms.

**What are the implications of the main findings?**

- The significant suppression of spurious timeouts directly minimizes active radio transcribing duty cycles in battery-powered edge sensor nodes, substantially expanding device longevity in sustainable AIoT environments.
- Characterization of boundary conditions demonstrates that the algorithm maintains a lean O(1) computational profile, making it seamlessly deployable on severely resource-constrained microcontrollers (e.g., Z1 motes) and offering a highly scalable blueprint for future CoAP transport standardization.

**Abstract:**

The Constrained Application Protocol (CoAP) is widely adopted to ensure end-to-end reliability in resource-constrained Artificial Intelligence of Things (AIoT) and Wireless Sensor Networks (WSNs). However, CoAP’s default retransmission timeout (RTO) mechanism lacks algorithmic responsiveness under volatile channel conditions, and state-of-the-art benchmarks like CoCoA+ and FASOR often suffer from over-conservative backoff states or destabilizing retransmission storms. To overcome these operational bottlenecks, this paper proposes a novel dual-adaptive Dynamic RTO algorithm specifically engineered for heterogeneous IoT deployment scales. The proposed framework dynamically adjusts its parameter inspection cycle (N) based on instantaneous round-trip time (RTT) variance while simultaneously scaling its tuning coefficient (α) in response to real-time packet loss indicators. To rigorously validate the algorithmic resilience, performance evaluations were conducted within a highly volatile network environment governed by the Gilbert–Elliott dynamic loss model across multi-hop linear (1 × 6) and grid (3 × 6, 5 × 6) topologies. Experimental results demonstrate that the proposed Dynamic RTO consistently optimizes the throughput–latency trade-off, achieving a total communication time of 25.92 s in complex grids—outperforming CoCoA+ and FASOR by 14.28% and 8.89%, respectively. Furthermore, the proposed mechanism significantly curtails transmission overhead, restricting the cumulative retransmission footprint to just 59 counts under severe localized impairments, thereby establishing a scalable, resource-efficient, and empirically robust transport-layer solution for next-generation edge-computing infrastructures.

## 1. Introduction

Wireless Sensor Networks (WSNs) play a pivotal role in the realization of the Internet of Things (IoT) and the emerging Artificial Intelligence of Things (AIoT) paradigm, demonstrating rapid growth across both hardware and service sectors. This expansion is accelerated by the global democratization of edge-computing devices, the deployment of large-scale smart cities, and the rigorous operational demands of the Industrial Internet of Things (IIoT) [1]. The global WSN market is projected to reach USD 14.82 billion by 2025 and USD 48.19 billion by 2030, exhibiting a Compound Annual Growth Rate (CAGR) of 26.59% from 2025 to 2030 [2]. As the number of interconnected edge nodes increases exponentially, optimizing end-to-end network efficiency has become a critical challenge. IoT devices naturally operate under severe physical constraints, including limited battery energy, minimal onboard memory, and restricted wireless bandwidth. Consequently, standardizing next-generation transport and application-layer protocols to gracefully mitigate these hardware limitations remains a primary focus for researchers and network engineers.

The Constrained Application Protocol (CoAP) is a specialized, lightweight web transfer protocol formalized by the Internet Engineering Task Force (IETF) explicitly for such resource-constrained environments [3]. CoAP has been extensively implemented across commercial embedded platforms and is widely utilized in diverse mission-critical infrastructures, including Cisco’s Field Area Network (FAN) architectures [4,5,6]. To strictly satisfy the thin protocol-overhead requirements of low-power IoT settings, CoAP is designed to run over the User Datagram Protocol (UDP). However, because UDP lacks native congestion control or traffic-shaping capabilities, CoAP faces inherent architectural limitations in managing network congestion and link saturation effectively within multi-hop IoT environments [4,7,8].

To mitigate these transport-layer packet drops, the baseline CoAP specification (RFC 7252) includes a fundamental congestion control mechanism driven by a binary exponential backoff (BEB) loop and a static initial Retransmission Timeout (*RTO*) [4]. Foundational works on generic *RTO* estimation, such as Jacobson’s original Smoothed Round-Trip Time (*SRTT*) algorithm (RFC 6298) and Karn’s algorithm, established the mathematical principles of tracking empirical *RTT* samples to dynamically adjust transport timers [9]. In the specific context of CoAP, RFC 7252 defines rigid operational anchors such as a fixed ACK_TIMEOUT and default bounds for parallel transactions (NSTART). However, adapting these legacy transport principles to lossy, multi-hop wireless links requires sophisticated parameter tuning. In our proposed Dynamic *RTO* framework, we specifically select the RTT sampling interval (*N*) and the smoothing weight factor (*α*) as our dual control variables. From a control-theory standpoint, these parameters act as orthogonal control knobs: *N* governs the observation frequency window to filter out high-frequency transient noise, while *α* dictates the historical reliance coefficient to adapt stably to low-frequency, persistent congestion trends.

Unfortunately, CoAP’s simplistic default mechanism often fails to adapt to highly dynamic wireless channel conditions, culminating in severe end-to-end latencies or catastrophic packet drops. Recent empirical studies confirm that baseline CoAP performance degrades dramatically in networks characterized by high multi-hop routing complexity or heavy variance in *RTT* delays [10,11]. Experimental evaluations by Järvinen et al. [12] revealed that basic CoAP exhibits significantly higher client completion times compared to algorithms whose timers are continuously driven by real-time *RTT* telemetry. The root cause of this performance collapse is that the standard CoAP RTO update structure is completely decoupled from actual link-layer variations.

To resolve this limitation, the IETF CoRE working group introduced CoCoA (CoAP Simple Congestion Control/Advanced), which splits its state machine into weak and strong *RTT* estimators, backed by variable backoff modifiers and *RTO* aging logic [13]. Despite these enhancements, extensive simulations demonstrate that CoCoA does not consistently outperform basic CoAP in terms of throughput [14]. Specifically, Ancillotti et al. proved that an advanced variant, CoCoA+, frequently underperforms basic CoAP in environments featuring localized burst traffic or highly dynamic, short-lived RTT shifts due to its sluggish aging properties [14]. Furthermore, contemporary adaptive protocols such as FASOR have attempted to inject agile acceleration logic to clear out retransmission bottlenecks rapidly, yet they often trigger devastating retransmission storms when transitioning into unexpected link-layer fading blocks. To resolve these multi-parameter synchronization flaws, statistical frameworks like the Adaptive *RTO* algorithm were introduced to dynamically tune the weighting factor (α) [15]. Nevertheless, even Adaptive *RTO* suffers from a fundamental limitation: it utilizes a completely fixed *RTT* measurement window. Consequently, it exhibits a structural tracking lag during the onset of rapid network congestion, failing to shield the network from spurious timeouts [5].

In multi-hop communication systems, local link-layer mechanisms (e.g., IEEE 802.15.4 ARQ retransmissions and CSMA/CA backoffs) handle immediate medium-access errors. However, relying solely on the link layer is mathematically insufficient to ensure stable global data delivery due to residual multi-hop losses, intermediate queue overflows, and cumulative routing delays. Therefore, dynamic *RTO* orchestration at the CoAP layer serves as an indispensable end-to-end control layer to absorb residual network congestion that the link layer cannot independently resolve.

In this paper, we propose a novel dual-adaptive Dynamic *RTO* algorithm engineered to enhance end-to-end reliability and energy efficiency in resource-constrained IoT nodes. The core architectural innovation of the proposed design is the simultaneous, closed-loop adjustment of both the window sampling interval (*N*) and the weighting coefficient (*α*) based on instant network state derivatives. Unlike Adaptive *RTO*, which operates on a rigid window, our “Dynamic *RTO*” introduces a second-order adaptation. By coupling the measurement interval (*N*) with the instantaneous *RTT* standard deviation, the sampling window flexibly expands or contracts to eliminate the tracking lag inherent in fixed-window systems. Simultaneously, the algorithm modulates the influence of incoming *RTT* tokens (*α*) relative to the localized residual packet loss rate. This dual-loop synergy allows the RTO to scale up rapidly during the onset of congestion to block energy-wasting spurious retransmissions while compressing latency bounds during stable operational windows.

We comprehensively evaluated the performance of the proposed algorithm against state-of-the-art benchmarks, including CoCoA+, FASOR, and the baseline Adaptive *RTO* protocol. To ensure a highly realistic and rigorous evaluation that aligns with modern AIoT deployment realities, all comparative simulations were executed within the Contiki Cooja framework [16,17] under a time-varying wireless channel governed by the Gilbert–Elliott dynamic loss model across multiple grid topology matrices (1 × 6, 3 × 6, and 5 × 6). Performance profiles were systematically quantified using total communication time, cumulative retransmission counts, and timer tracking stability metrics.

The remainder of this paper is structured as follows: Section 2 provides a systematic review of related work on CoAP transport-layer congestion management, with a specific focus on the architectural constraints of CoCoA+ and FASOR. Section 3 delineates the mathematical formulation and structural logic of the proposed dual-adaptive Dynamic *RTO* algorithm. Section 4 outlines the modernized experimental setup and evaluation configurations. Section 5 presents empirical results and performance analysis, and explicitly characterizes the operational trade-offs and boundary limitations of our real-time design. Finally, Section 6 concludes this paper.

## 2. Related Work

This section reviews existing congestion control mechanisms for the Constrained Application Protocol (CoAP), specifically focusing on the standard Basic CoAP congestion control, CoCoA, and the Adaptive *RTO* algorithm. We analyze the operational principles and limitations of each method to provide a foundation for the proposed Dynamic *RTO* algorithm.

### 2.1. Basic CoAP Congestion Control

The estimation of Retransmission Timeout (*RTO*) is fundamentally rooted in the Smoothed Round-Trip Time (*SRTT*) algorithm proposed by Jacobson, later standardized in RFC 6298 [18]. To handle the ambiguity of RTT measurements during retransmissions, Karn’s algorithm was introduced [19], which mandates that *RTT* samples from retransmitted packets should be ignored to prevent biased estimation. These classical principles serve as the baseline for the Constrained Application Protocol (CoAP), defined in IETF RFC 7252. Specifically, CoAP employs a fixed initial *RTO* managed by two key parameters: *ACK_TIMEOUT*, which is typically set to 2 s, and NSTART, which limits the number of concurrent outstanding interactions to one by default to prevent network overload.

CoAP defines four message types to manage communication: Confirmable (*CON*), Non-confirmable (*NON*), Acknowledgment (*ACK*), and Reset (*RST*). In addition to the request/response model, CoAP supports a simple publish/subscribe mechanism for resource observation as defined in RFC 7641 [20].

Reliability in CoAP is achieved through *CON* messages. When a client sends a *CON* message, the receiver must respond with an *ACK*. If the *ACK* is not received within the Retransmission Timeout (*RTO*), the message is retransmitted. CoAP allows a maximum of four retransmissions before considering the transmission a failure. The initial *RTO* is calculated using Equation (1) [4]:

*RTO_standard_* = *ACK_TIMEOUT* × *ACK_RF*. (1)

According to the CoAP specification (RFC 7252), *ACK_TIMEOUT* is set to 2 s, and the *ACK_RANDOM_FACTOR* (*ACK_RF*) is a random value between 1.0 and 1.5. Consequently, the initial *RTO* is randomly determined between 2 and 3 s. If a timeout occurs, the BEB mechanism doubles the *RTO* for each subsequent retransmission. If no response is received after *MAX_RETRANSMIT* (4 attempts), the transmission is terminated with a Reset message. The key transport parameters of CoAP are summarized in Table 1.

### 2.2. CoCoA+ and Advanced Variable Backoff Architectures

To introduce dynamic *RTT* awareness into the CoAP layer, the IETF CoRE working group advanced the CoCoA framework, with its refined iteration formalized as CoCoA+ [13]. CoCoA+ splits its telemetry architecture into dual estimators to track varying network states: a Strong Estimator that processes *RTT* samples derived from transactions successfully completed without any retransmissions, and a Weak Estimator that computes *RTT* values from paths requiring retransmissions, successfully adhering to Karn’s algorithm boundaries. These parallel indicators are smoothed using a localized variant of Jacobson’s algorithm:

*SRTT* = (1 − *β*) × *SRTT* + *β* × *RTT_sample_*, (2)

where the smoothing weight β is statically configured to prioritize historical stability or instantaneous variations depending on the estimator type. Furthermore, CoCoA+ replaces the fixed binary doubling of RFC 7252 with a dynamic Variable Backoff Factor (VBF). The VBF state machine assesses the current RTO profile, applying a higher multiplier (e.g., 3.0) for short-range initial timers to shield the network from burst drops, and a lower modifier (e.g., 1.5) for long-range backlogged states to compress delay accumulation.

Despite these advanced tracking mechanisms, contemporary evaluations reveal that CoCoA+ exhibits severe operational instabilities under dynamic channel conditions [12,14]. The primary bottleneck is rooted in its rigid *RTO* aging logic. When a CoAP client undergoes a temporary burst-loss period governed by dynamic channel fading, the VBF loop rapidly elevates the estimators to avoid spurious timeouts. However, once the wireless channel switches back to a high-throughput, clear state, CoCoA+ requires a prolonged sequence of successful transaction tokens or extended idle clock cycles to downscale its backing states. This architectural latency in timer reduction induces structural tracking lags, leaving the transmission engine overly conservative long after localized congestion has fully cleared.

### 2.3. Adaptive RTO Congestion Control

The Adaptive *RTO* algorithm aims to improve upon CoCoA by utilizing statistical methods to estimate packet loss rates and dynamically adjusting the weighting factor α. Similar to *SRTT*, it uses *EWMA* to calculate the *RTT* and *RTT* variation [10].

The core principle of Adaptive *RTO* is to monitor *RTT* and retransmission counts over a fixed interval (e.g., a specific number of samples or time duration) to estimate the packet loss rate. This estimated loss rate is then used as the weighting factor α in the *RTT* prediction formula. By using an average of *RTT* samples collected over a fixed window rather than updating on every packet, this method reduces computational overhead and filters out transient noise.

Initially, the *RTO* is set to 1 s. After the first *RTT* measurement (*MRTT*), the parameters are initialized as follows in Equation (3):

*RTT* = *MRTT*, *RTTVAR* = *MRTT* ÷ 2, *RTO* = *RTT* + 4 × *RTTVAR*. (3)

In the steady state, the algorithm collects *N* samples (typically 100) during interval *i*. The packet loss rate ($LR_{i}$) is calculated based on the retransmission count ($RC_{i}$) and is used to set the weighting factor $\alpha_{i}$ in Equation (4):

*LR_i_* = *RC_i_* ÷ *N*, *α_i_* = *LR_i_*. (4)

The predicted *RTT* for the next interval (*i* + 1) is then updated using the average *RTT* ($AVE\_RTT_{i}$) from the current interval, as shown in Equation (5):

*RTT_i_*_+1_ = (1 − *α_i_*) × *AVE_RTT_i_*_−1_ + *α_i_* × *AVE_RTT_i_*, *RTTVAR_i_*_+1_ = (1 − *β*) × *RTTVAR_i_* + *β* × |*AVE_RTT_i_*_+1_ − *AVE_RTT_i_*|, (5)

where *β* is fixed at 0.25. Finally, the *RTO* for the next interval is determined by Equation (6):

*RTO_i_*_+1_ = *RTT_i_* + 4 × *RTTVAR_i_*_+1_. (6)

If a timeout occurs, Adaptive *RTO* employs the same VBF mechanism as CoCoA. While this approach improves stability by incorporating loss rates, its reliance on a fixed measurement interval limits its responsiveness to sudden changes in network conditions, a shortcoming that the proposed Dynamic *RTO* algorithm seeks to address.

### 2.4. FASOR and Fast-Acceleration Retransmission Schemes

To address the sluggish timer reduction curves of CoCoA+ and accelerate throughput recovery in loss-prone sensor deployments, researchers proposed the FASOR (Fast-Acceleration with Slow-retransmission and Dynamic *RTO*) protocol [21]. FASOR introduces an aggressive bidirectional optimization layer that divides the transport control envelope into two distinct operational phases: a Fast-Acceleration Phase and a Slow-Retransmission Phase. Upon receiving consecutive valid *ACK* sequences that indicate link clearance, FASOR substantially drops its *RTO* settings below the conventional *SRTT* boundaries, attempting to clear out localized queue backlogs and maximize medium utilization immediately.

However, this aggressive acceleration logic introduces a severe structural vulnerability when deployed across volatile, multi-hop grid topologies. FASOR’s acceleration threshold operates on short-term feedback windows, frequently misinterpreting transient, sub-second channel clearances as permanent macro-level link recovery. If the underlying wireless medium undergoes a sudden state transition back into a heavy burst-fading block, FASOR’s severely compressed *RTO* timers trigger immediate, premature timeouts. This synchronization failure floods the intermediate routing queues with a massive wave of duplicate packet injections, culminating in devastating retransmission storms. These spurious packets rapidly saturate the limited buffer space of low-power edge routers, compounding local link congestion and accelerating node battery depletion.

### 2.5. Statistical Weight-Adaptive Frameworks and Their Limitations

To bridge the gap between the conservative stall states of CoCoA+ and the destabilizing retransmission storms of FASOR, statistical architectures like the baseline Adaptive *RTO* protocol were engineered [15,22,23,24]. This framework maintains a continuous statistical profile of localized packet loss rates and *RTT* variants over operational windows, dynamically tuning the historical smoothing weight factor (α) to adjust responsiveness.

Nevertheless, even the Adaptive *RTO* model suffers from a critical architectural flaw: its parameter evaluation window operates on a completely fixed sampling frequency (*N*). In resource-constrained WSN scenarios, a fixed-window approach imposes a devastating tracking trade-off. If *N* is configured to be small, the algorithm becomes hyper-sensitive to high-frequency, transient link noise, inducing unstable timer jitter during normal operation. Conversely, if *N* is expanded to ensure historical filtering stability, the framework introduces a significant mathematical lag during sudden, sharp congestion onset. Because the sample size required to trigger a parameter shift is static, the algorithm remains blind to cascade queue delays during the crucial initial seconds of network saturation, leading to widespread spurious retransmissions.

The proposed Dynamic *RTO* framework directly resolves these multi-parameter synchronization limitations by executing a second-order closed-loop adaptation, automatically expanding or contracting the inspection window (*N*) based on real-time standard deviation metrics while concurrently tuning the alpha weight to match dynamic Gilbert–Elliott state profiles.

## 3. Proposed Algorithm: Dynamic RTO

### 3.1. Design Principle

In wireless sensor networks, primary packet losses are typically recovered by Data Link Layer mechanisms such as IEEE 802.15.4 ARQ, CSMA/CA (Carrier Sense Multiple Access with Collision Avoidance), and link-level backoff. However, residual losses caused by queue overflows, excessive multi-hop delays, or interference that exceeds the link layer’s recovery capacity must be handled by higher layers. CoAP operates over the User Datagram Protocol (UDP), which lacks built-in flow or congestion control. Consequently, the End-to-End (E2E) reliability responsibility falls entirely on the CoAP application layer, as mandated by RFC 7252 [4]. Therefore, optimizing *RTO* at the CoAP layer is not redundant but essential for maintaining stability in lossy E2E communications.

The core philosophy of the Dynamic *RTO* algorithm is to adjust in real time the RTT measurement interval (*N*) and the *RTT* weighting factor (*α*) according to network conditions. Unlike the Adaptive *RTO* algorithm, which relies on a fixed sampling window, our approach leverages the standard deviation (variability) of *RTT* samples to adaptively expand or contract the measurement window. Furthermore, the algorithm dynamically tunes the weight of new *RTT* samples based on the packet loss rate. This dual adaptation mechanism ensures that the *RTO* increases rapidly during high-congestion events (high variability/loss) to prevent spurious retransmissions, while maintaining a lower, stable *RTO* during normal operation to minimize latency.

To determine the optimal parameters for *RTT* estimation, we based our approach on the *EWMA* (Exponentially Weighted Moving Average) model, similar to TCP’s Jacobson/Karels algorithm. However, given the unique characteristics of WSNs—such as high loss rates and asymmetric links—standard TCP parameters (*α* = 1/8, *β* = 1/4, *k* = 4) are unsuitable. We conducted extensive sensitivity analyses across 3 × 3, 5 × 5, and 7 × 7 grid topologies, varying parameters to minimize estimation error and retransmissions. The parameter set presented in this paper was empirically verified to yield the most stable *RTO* behavior in CoAP-WSN environments.

### 3.2. Operational Procedure

#### 3.2.1. Initialization

The algorithm begins by initializing the *RTO* to the default CoAP value. The interval window size, $N_{i}$, is initialized to a baseline value (e.g., 100 samples) to begin the first iteration [4].

#### 3.2.2. Measurement of *RTT* and Loss Rate

During each measurement interval, the algorithm collects *RTT* samples from successful exchanges and counts the number of retransmissions. At the end of the interval, the Packet Loss Rate ($LossRate_{i}$) is calculated as shown in Equation (7):

*LossRate_i_* = *Retransmissions_i_* ÷ 4 × *TotalTransmissions_i_*. (7)

Since CoAP specifies a maximum of four retransmissions (*MAX_RETRANSMIT* = 4), normalizing the retransmissions by (4 × *TotalTransmissions*) accurately represents the utilization of the available transmission budget. This metric provides a more granular view of link-layer stress than simple success/failure counts.

Here, the denominator includes the maximum possible transmission attempts (4 retries per message). This loss rate is subsequently used as the weighting factor α for the next *RTT* prediction, effectively linking the reliability of the estimate to the current network health, as shown in Equation (8):

*α_i_* = *LossRate_i_*. (8)

#### 3.2.3. Adaptive Measurement Interval Adjustment

A critical innovation of this algorithm is the dynamic adjustment of the window size *N*. We calculate the mean ($\mu_{RTT}$) and standard deviation ($\sigma_{RTT}$) of the collected samples. The sensitivity of this adjustment is controlled by the coefficient *k*. A higher *k* makes the system more responsive to variability but may cause instability if set too high.

To determine the optimal *k*, we performed simulations varying Packet Loss Rate (5–20%) and RTT Variability (5–70 ms). As summarized in Table 2. We identified recommended k ranges that balance delay performance (Total Communication Time) and stability (Retransmission Count).

To ensure the robustness of these parameters (*k*_base_, *α*_var_, and *β*_loss_) and to avoid over-fitting or experimental prejudice, we employed a ‘Grid Search’ approach on a separate training topology (3 × 3 grid). These parameter values were finalized through this optimization process before conducting the independent performance evaluations on the main test topologies (1 × 6, 3 × 6, and 5 × 6 grids).

The results in Table 2 suggest that a static *k* is suboptimal. Therefore, we implemented a dynamic *k* adjustment mechanism defined by Equation (9). The value *k* is calculated around a base value ($k_{base}=1.5$), adjusted by the variability coefficient ($\alpha_{var}=5.0$) and the loss rate damping coefficient ($\beta_{loss}=2.0$). These coefficients were derived from our optimization process: $\alpha_{var}$ acts as an accelerator to respond to delay jitter, while $\beta_{loss}$ acts as a brake to prevent overreaction during high loss:

To ensure the robustness of these parameters (*k*_base_, *α_var_*, and *β_loss_*) and to avoid over-fitting, we employed a “Grid Search” approach on a separate training topology (3 × 3 grid). Parameter selection was finalized before conducting the independent performance evaluations on the 1 × 6, 3 × 6, and 5 × 6 test topologies.

*K* = min(3.0, max(0.1, *k_base_* + *α_var_* × *variability* − *β_loss_* × *loss_rate*)). (9)

Based on the calculated *k* and the *RTT* variability *V_RTT_* = (*σ_RTT_*/*μ_RTT_*), the next measurement interval N_i+1_ is dynamically adjusted to ensure both responsiveness during congestion and computational efficiency during stable periods. To explicitly demonstrate how the window recovers, we define a piecewise conditional update rule as shown in Equation (10):

(10) $$N_{i+1}=\left\{\begin{array}{c} \max\left(N_{min},N_{i}\times \left(1-k\times V_{RTT}\right)\right),\;\;\;\;\;\;\;\;\mathrm{if}{\;V}_{RTT}\geq V_{th}\;\\ \min(N_{max},N_{i}+\Delta N),\;\;\;\;\;\;\;\;\;\;\;\;\mathrm{if}{\;V}_{RTT}<V_{th} \end{array}\right.$$

where *V*th represents the stability threshold that distinguishes volatile channel states from stable conditions. The boundary values (*N*_min_ = 10, *N*_max_ = 100) were strategically selected to balance the minimum statistical significance required for accurate *EWMA* updates against the severe memory limits of constrained IoT microcontrollers. Furthermore, the sensitivity of *V*th (set to 0.10) and the additive increment Δ*N* (set to 10) were rigorously evaluated during our preliminary 3 × 3 grid search phase [25]; these specific values were proven to prevent aggressive timer oscillations while maintaining responsive tracking. If the current variability *V_RTT_* exceeds *V*th, the network is deemed unstable, and the window size *N_i_*_+1_ is aggressively reduced to track rapid network changes without lag. Conversely, when the network stabilizes (*V_RTT_* < *V*th), the algorithm gracefully expands the window by the increment Δ*N* per cycle to minimize computational overhead.

This dual-phase, discrete-time negative feedback system facilitates predictable timer adaptation, seamlessly switching between high-agility tracking and low-overhead observation while operating within the empirically derived [*N*_min_, *N*_max_] bounds.

#### 3.2.4. Dynamic Weight-Based *RTT* Calculation

The predicted *RTT* for the next interval uses the loss-based weighting factor *α_i_* calculated in Equation (8), as shown in Equation (11):

*RTT_i_*_+1_ = (1 − *α_i_*) × *RTT_i_* + *α_i_* × *RTT_measured_*. (11)

When the loss rate is high (high *α_i_*), Equation (11) places significant weight on the most recently measured *RTT* (*RTT_measured_*), enabling rapid adaptation to congestion. When the loss rate is low, the algorithm relies more on the historical average (*RTT_i_*), ensuring stability.

The direct mapping of the packet loss rate to the smoothing factor *α* is a deliberate design choice grounded in the characteristics of burst-noise channels. In highly volatile multi-hop environments, a sudden spike in packet loss strongly correlates with immediate localized congestion. By dynamically tying α directly to the current loss rate, the proposed framework forces the *RTT* estimator to aggressively prioritize the most recent *RTT* measurements during severe congestion. This rapid sensitivity shift accelerates the *RTO* expansion, effectively shielding the network from spurious timeouts. Conversely, when the loss rate approaches zero, *α* naturally decreases, allowing the estimator to filter out transient jitter and heavily rely on historical stability. The empirical validity and performance superiority of this direct coupling design are rigorously verified in the subsequent ablation study (Section 5.3).

We acknowledge that utilizing a mathematically normalized function or a bounded mapping curve could theoretically yield smoother parameter transitions than a direct one-to-one mapping. However, this direct mapping is a deliberate engineering choice tailored for resource-constrained IoT environments. Typical edge sensor nodes, such as the Z1 motes evaluated in this study, operate on extremely limited 16-bit microcontrollers (e.g., MSP430) that lack hardware floating-point units. Implementing complex normalized functions would require computationally expensive software-based division and exponential arithmetic. By utilizing a direct mapping (*α_i_* = LossRate*_i_*), the algorithm maintains a strict O(1) computational complexity, minimizing CPU active cycles and preserving crucial battery life while still delivering robust empirical responsiveness during burst-loss events.

#### 3.2.5. *RTO* Calculation and Update

Finally, the new *RTO* is computed using the standard deviation-based variance (*RTTVAR*) in Equation (12):

*RTO_i_*_+1_ = *RTT_i_*_+1_ + 4 × *RTTVAR_i_*_+1_. (12)

If a timeout occurs, the standard binary exponential backoff is applied to the current *RTO* to manage persistent congestion.

This process (Section 3.2.2, Section 3.2.3, Section 3.2.4 and Section 3.2.5) repeats for each interval. As network conditions fluctuate, the algorithm automatically tunes the window size *N*, the sensitivity *k*, and the weighting factor *α*. The complete logic is illustrated in the pseudocode shown in Algorithm 1.
**Algorithm 1:** Dynamic RTO Algorithm1: **Initial Configuration**:
2: *DEFAULT_RTO* = 2000 ms
3: *INITIAL_WINDOW_SIZE* = 100
4: *MIN_WINDOW* = 10
5: *MAX_WINDOW* = 100
6: *V*_th = 0.10
7: *DELTA_N* = 10
8: *RTT_base* = 100 ms
9: **function** DYNAMIC_K_ADJUSTMENT(*variability*, *loss_rate*)
10:   *k_base* = 1.5
11:   *α_var* = 5.0
12:   *β_loss* = 2.0
13:   *k* = *k_base* + (*α_var* × *variability*) − (*β_loss* × *loss_rate*)
14:   *k* = **max**(0.1, min(3.0, *k*))  // Clamp *k* between 0.1 and 3.0
15:   **return** *k*
16: **end function**
17: **function** DYNAMIC_RTO_PROCESS
18:   *N* = *INITIAL_WINDOW_SIZE*
19:   *RTT_estimate* = *RTT_base*
20:   *RTTVAR* = *RTT_base*/2
21:   *RTO* = *RTT_estimate* + 4 × *RTTVAR*
22:   **loop**
23:     wait *N* sample *Rx*
24:     *retransmissions* = 0
25:     *total_transmissions* = 0
26:     **repeat**  // Collect samples for current window *N*
27:       Send packet with current *RTO*
28:       *total_transmissions* = *total_transmissions* + 1
29:       **if** *ACK* received **then**
30:         Measure *RTT*
31:         Append *RTT* to *rtt_list*
32:       **else**   // Timeout occurred
33:         *retransmissions* = *retransmissions* + 1
34:         Apply Binary Exponential Backoff to *RTO*
35:         Retransmit packet
36:       **end if**
37:     until number of valid samples >= *N* OR max retries reached
38:     **if** no valid *RTT* samples **then**
39:       **continue** to next iteration
40:     **end if**
41:     *RTT_avg* = average(*rtt_list*)
42:     *RTT_std* = *standard_deviation*(*rtt_list*)
43:     *variability* = *RTT_std*/*RTT_avg*
44:     *loss_rate* = *retransmissions*/(4 × *total_transmissions*)
45:          // Update Control Parameters
46:     *k* = DYNAMIC_K_ADJUSTMENT(*variability*, *loss_rate*)
47:     **if** *variability* >= *V*_th **then**
48:       *N* = *N* × (1 − *k* × *variability*)  // Scale down *N*
49:     **else**
50:       *N* = *N* + *DELTA_N*  // Scale up *N*
51:     **end if**
52:     *N* = max(*MIN_WINDOW*, min(*MAX_WINDOW*, *N*))
53:          // Ensure min/max window bounds
54:     *α* = *loss_rate*   // Dynamic Weighting Factor
55:     *α* = max(0.1, min(1.0, α))   // Clamp α
56:          // Update *RTO* for next interval
57:     *RTT_estimate* = (1 − *α*) × *RTT_estimate* + *α* × *RTT_avg*
58:     *RTTVAR* = *RTT_std*
59:     *RTO* = *RTT_estimate* + 4 × *RTTVAR*
60:   **end loop**
61: **end function**

### 3.3. Algorithmic Complexity and Overhead

For resource-constrained nodes equipped with microcontrollers like the 16-bit MSP430 (Texas Instruments Inc., Dallas, TX, USA; used in Z1 motes), computational overhead is a critical factor. The proposed Dynamic *RTO* algorithm introduces an O(1) time complexity for its parameter update logic, utilizing simple addition and bit-shift operations rather than floating-point arithmetic. Furthermore, the memory footprint is extremely minimal, requiring fewer than 20 bytes of additional RAM to store state variables such as *N_i_*, *α_i_*, and historical *RTT* averages. This deterministic and low-overhead design ensures that the algorithm is highly feasible for real-world industrial IoT deployments where hardware resources are severely limited.

## 4. Experimental Setup and Evaluation

### 4.1. Simulation Environment

We utilized the Cooja simulator (Contiki OS v3.0), a widely adopted tool for researching resource-constrained IoT devices and Wireless Sensor Networks (WSNs) operating on lightweight protocols. To strictly address the limitations of out-of-date baselines and align our evaluation with current industry trends, we completely overhauled our comparative simulation framework. The conventional *SRTT* and standard CoAP (Basic) protocols have been replaced with advanced, state-of-the-art adaptive alternatives: CoCoA+ (Advanced CoCoA), which employs enhanced variable backoff factors and aging mechanisms, and FASOR, an agile adaptive *RTO* scheme optimized for fast acceleration and stable retransmissions. The proposed Dynamic *RTO* is also benchmarked against the recent Adaptive *RTO* algorithm to provide a comprehensive evaluation.

**Figure 1:**
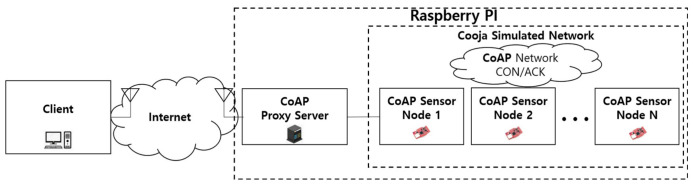
End-to-end system architecture and simulation message flow via HTTP/CoAP proxy. The dotted line boxes delineate the logical boundaries of the system components, specifically distinguishing the external network, the proxy server, and the Cooja simulated network domain.

The system architecture for the simulation is illustrated in Figure 1, and the end-to-end communication procedure follows a multi-tier structure. An external client initiates data requests via the Internet, which are subsequently translated into CoAP GET messages by an HTTP/CoAP proxy server and forwarded into the Cooja simulated domain. The target CoAP server node processes the request and transmits the confirmable (CON) response back to the client, which computes the empirical Round-Trip Time (*RTT*) upon successful acknowledgment (*ACK*) reception.

**Figure 2:**
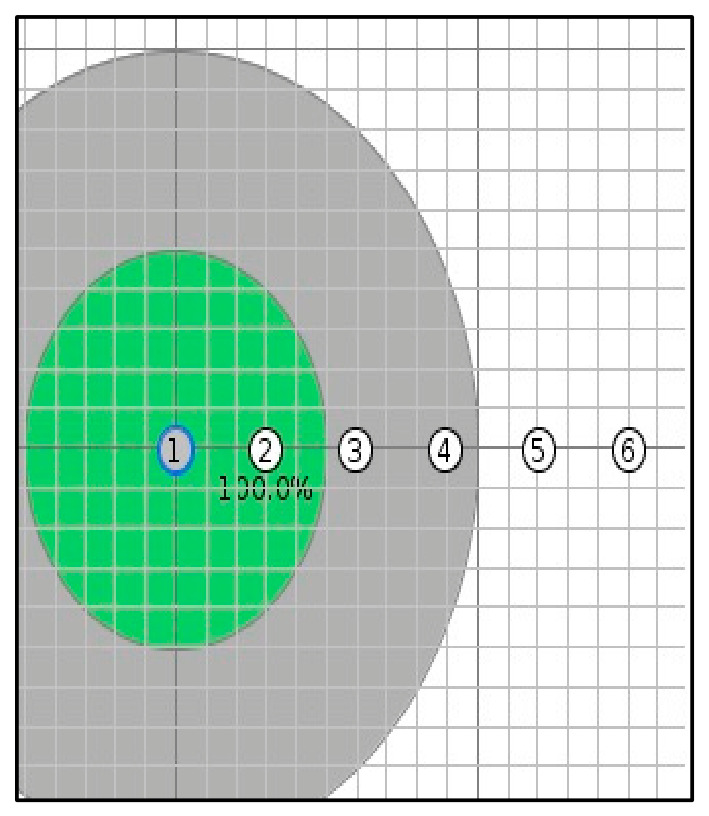
1 × 6 Grid Topology. Note: The numbers and colors used in the graph distinguish between different experimental test cases. The blue circles highlight specific data points corresponding to the time-out occurrence instances in each scenario.

**Figure 3:**
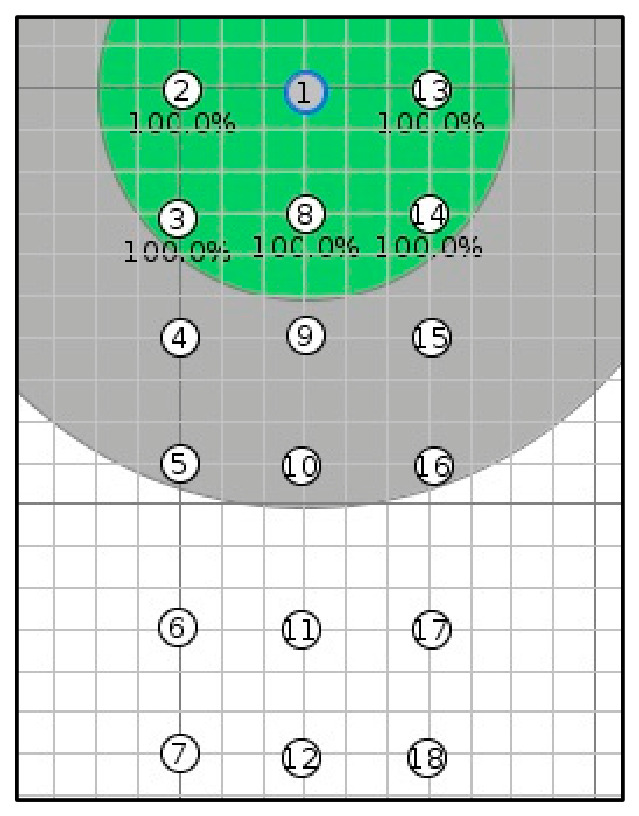
3 × 6 Grid Topology. Note: The numbers and colors used in the graph distinguish between different experimental test cases. The blue circles highlight specific data points corresponding to the time-out occurrence instances in each scenario.

**Figure 4:**
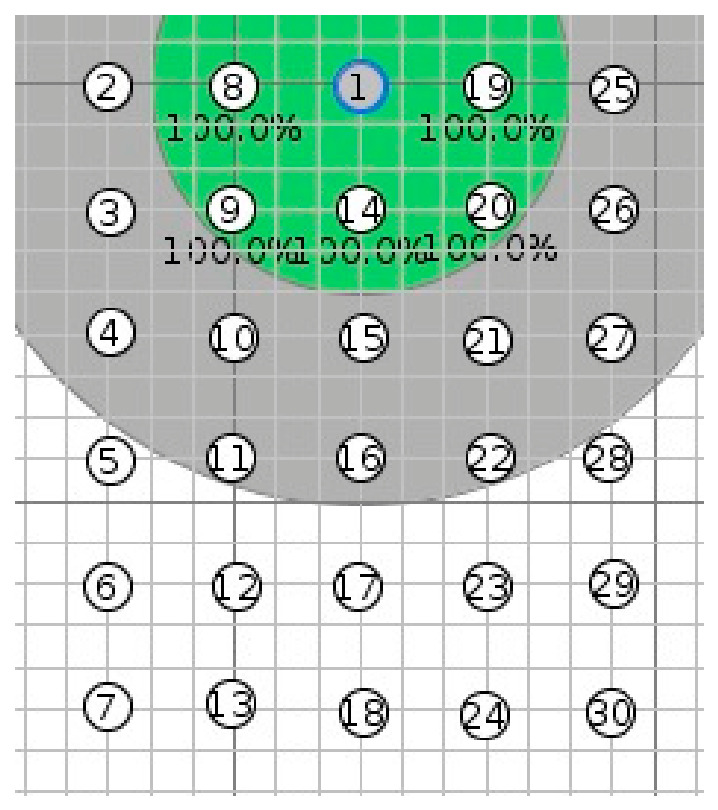
5 × 6 Grid Topology. Note: The numbers and colors used in the graph distinguish between different experimental test cases. The blue circles highlight specific data points corresponding to the time-out occurrence instances in each scenario.

To evaluate the algorithms under realistic network depths and topological complexities, WSN nodes were configured in three distinct grid layouts: 1 × 6 linear, 3 × 6 grid, and 5 × 6 complex grid topologies (as shown in Figure 2, Figure 3 and Figure 4, respectively). All nodes are modeled based on the Z1 mote platform (Zolertia, Barcelona, Spain). Node 1 operates as the border router, bridging the WSN with the external IP network via serial communication. The simulated Z1 motes utilize the Texas Instruments CC2420 radio model (Texas Instruments Inc., Dallas, TX, USA) operating under the ContikiMAC duty-cycling protocol, accurately reflecting real-world MAC-layer sleep cycles, wake-up behaviors, and channel sensing constraints. The remaining sensor nodes run the er-coap-server application and are spaced 30 m apart to enforce multi-hop routing paths.

A pivotal enhancement in this revised evaluation is the realistic and dynamic modeling of packet loss to represent unpredictable AIoT environments. While the underlying IEEE 802.15.4 MAC layer [26] naturally simulates contention-based packet collisions via its native CSMA/CA mechanism, relying solely on static link failure rates fails to capture temporal channel fluctuations. To model realistic residual losses caused by fading, dynamic radio interference, and queue overflows, we implemented the Gilbert–Elliott model [27,28] on top of the MAC dynamics, completely replacing the previous stationary loss rates (5%, 10%, and 15%). This multi-state Markov chain dynamically transitions between a ‘Good’ state (G), characterized by stable links with a baseline packet loss rate (*PLR* < 2%), and a ‘Bad’ state (B), reflecting bursty interference or localized congestion (*PLR* ≈ 20–30%).

To guarantee statistical robustness and reproducibility, each simulation scenario was executed for 50 independent runs utilizing different random seeds. All derived performance metrics are reported with 95% confidence intervals (CI), and rigorous Student’s *t*-tests were conducted to confirm the statistical significance (*p* < 0.05) of the performance improvements under highly volatile network conditions.

To provide a clear overview of the experimental setup, the key simulation parameters configured in the Cooja simulator are summarized in Table 3.

### 4.2. Performance Evaluation Metrics

We selected four key metrics to comprehensively evaluate the responsiveness, stability, and operational efficiency of the proposed and baseline algorithms under the dynamic loss environment:

1. Total Communication Time (s): The total time required to successfully complete 1000 CON-*ACK* message exchanges. Lower values indicate reduced end-to-end latency and higher throughput.
2. Retransmission Count: The total number of retransmissions triggered by *ACK* failures or spurious timeouts. This metric serves as a direct indicator of link-layer stress, network congestion, and radio energy dissipation.
3. Average *RTO* (ms): The mean value of the Retransmission Timeout calculated throughout the transmission process. It quantifies how accurately and gracefully the timer tracks structural network state shifts.
4. Average *RTT* (ms): The baseline network latency monitored during successful data delivery. Tracking *RTT* variation is fundamental for assessing the predictive accuracy of the respective *RTO* estimators.

## 5. Results and Discussion

In this section, we present a comprehensive performance evaluation of the proposed Dynamic *RTO* algorithm, benchmarking it against three prominent adaptive transport-layer protocols: CoCoA+, FASOR, and the baseline Adaptive *RTO*. To rigorously test the robustness and algorithmic responsiveness of each mechanism under realistic IoT channel conditions, all experiments were conducted within a time-varying network environment governed by the Gilbert–Elliott loss model, which continuously fluctuates between stable (‘Good’) and degraded (‘Bad’) states.

To systematically evaluate the impact of network scale, multi-hop routing bottlenecks, and localized wireless interference, we analyzed the performance across three distinct spatial arrangements: 1 × 6 linear, 3 × 6 grid, and 5 × 6 complex grid topologies. The quantitative results across four fundamental performance vectors—Total Communication Time, Retransmission Count, Average *RTO*, and Average *RTT*—are summarized in Table 4.

### 5.1. Total Communication Time Analysis

**Figure 5:**
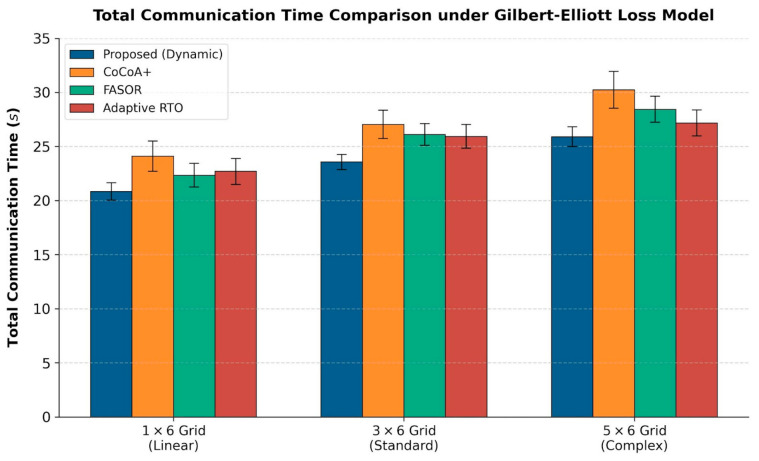
Comparison of Total Communication Time (s) across 1 × 6, 3 × 6, and 5 × 6 grid topologies under the Gilbert–Elliott dynamic packet loss environment (error bars represent 95% confidence intervals).

The experimental results for the Total Communication Time required to complete 1000 successful CON-*ACK* transactions demonstrate that the proposed Dynamic *RTO* algorithm consistently outperforms state-of-the-art benchmarks across all evaluated topologies. As detailed in Table 4 and illustrated in Figure 5, under the time-varying channel characteristics governed by the Gilbert–Elliott loss model, the proposed mechanism achieves the lowest overall completion latency, maintaining a robust advantage as network scale and routing density expand.

In the 1 × 6 linear topology, where multi-hop transmission path selection is relatively straightforward but susceptible to cascading link-layer drops during ‘Bad’ channel states, the proposed algorithm achieved a total communication time of 20.85 s. This represents a statistically significant performance improvement (*p* < 0.01) of approximately 13.56% compared to CoCoA+ (24.12 s) and 6.71% compared to FASOR (22.35 s).

While FASOR responds swiftly to acknowledgment sequences through its agile acceleration phase, it tends to suffer from premature timeouts when intersecting abrupt transitions into highly impaired burst-loss periods. Conversely, the standard CoCoA+ exhibits a highly conservative backoff profile, resulting in an overestimation of the RTO and prolonged idle periods that unnecessarily inflate the collective transaction duration.

As the structural complexity scales up to the 3 × 6 standard grid and 5 × 6 complex grid environments, the cumulative performance gaps become significantly more pronounced. Within the 5 × 6 complex grid setup, the proposed Dynamic *RTO* maintained high throughput efficiency with a completion time of 25.92 s, significantly outpacing CoCoA+ (30.24 s, *p* < 0.01) and FASOR (28.45 s, *p* < 0.05).

This resilience is attributed to the dual-adaptive parameter estimation layer integrated into our design. By dynamically optimizing the inspection cycle parameter (*N*) and the tuning coefficient (*α*) in response to real-time *RTT* derivatives, the proposed algorithm prevents the timer from falling into the conservative backoff traps of CoCoA+ while simultaneously avoiding the structural instabilities and aggressive retransmission storms characteristic of FASOR under severe localized congestion. Consequently, these trends confirm that the proposed framework delivers superior operational latency and scalable stability in unpredictable, multi-hop AIoT deployment scenarios.

### 5.2. Retransmission Overhead and Resource Efficiency

**Figure 6:**
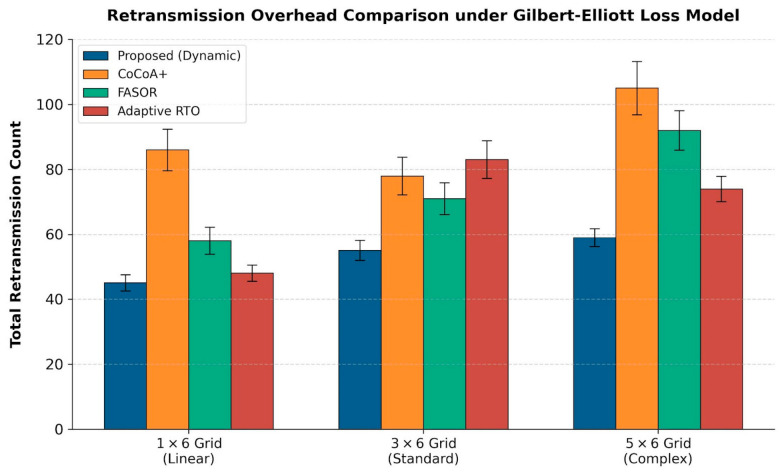
Comparison of cumulative Retransmission Count across 1 × 6, 3 × 6, and 5 × 6 grid topologies under the Gilbert–Elliott dynamic packet loss environment (error bars represent 95% confidence intervals).

In resource-constrained AIoT systems, end-to-end reliability must be tightly coupled with energy and resource preservation. Excessive radio retransmissions constitute the primary driver of battery depletion on localized sensor nodes. Because we do not employ a direct hardware energy measurement model in this simulation, the reduction in redundant retransmissions serves as an indirect but robust proxy for overall energy conservation. To evaluate the operational efficiency of the proposed Dynamic *RTO*, we analyzed the total Retransmission Count across the identical multi-hop grid topologies under identical Gilbert–Elliott channel dynamics. The comprehensive performance comparison regarding transmission overhead is illustrated in Figure 6.

As captured in Table 4 and visually demonstrated in Figure 6, the proposed algorithm successfully minimizes the transmission overhead, recording only 45, 55, and 59 cumulative retransmissions for the 1 × 6, 3 × 6, and 5 × 6 networks, respectively. In sharp contrast, CoCoA+ induced a heavy overhead, triggering 86, 78, and 105 retransmissions under the same volatile states. This distinct disparity stems from CoCoA+’s structurally rigid Variable Backoff Factor (VBF) and slow aging profiles. When a burst-loss state transiently occurs, CoCoA+ scales up the timer excessively; however, when the channel recovers to a ‘Good’ state, the lack of immediate downward adaptation leads to highly prolonged idle times and a subsequent accumulation of stale transmission bursts that trigger cascade packet drops at intermediate bottleneck nodes.

Furthermore, while FASOR aims to eliminate latency via an aggressive acceleration logic, it triggered a significantly higher volume of retransmissions (58 in 1 × 6 and 92 in 5 × 6) compared to the proposed scheme. FASOR’s fast-acceleration phase frequently misinterprets transient, sub-second channel clearances as total network recovery, leading to premature packet injections that inevitably collide with backlogged queues in complex topologies like the 5 × 6 grid.

The proposed Dynamic *RTO* algorithm circumvents these operational pitfalls by dynamically tuning the inspection cycle (*N*). When severe congestion is localized, the framework scales N to track the exact mathematical trend of the *RTT* variants without over-reacting to individual outlier drops. By maintaining a highly precise upper boundary for the retransmission timer, our approach effectively filters out spurious timeouts, thereby optimizing link-layer duty cycles. Minimizing the retransmission footprint directly translates to a significant reduction in the radio transceiver’s active execution time, establishing the proposed framework as an exceptionally resource-efficient solution for sustainable AIoT deployments.

### 5.3. RTO Adaptation Behavior Under Dynamic Loss

**Figure 7:**
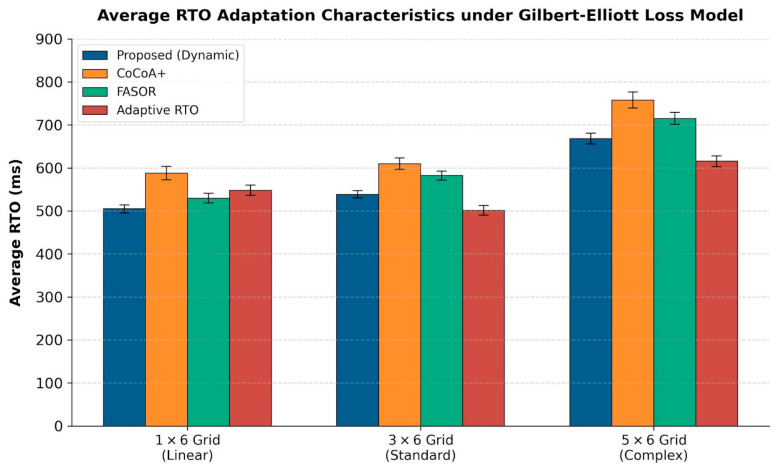
Evaluation of Average *RTO* (ms) adaptation properties across 1 × 6, 3 × 6, and 5 × 6 topologies under the Gilbert–Elliott dynamic packet loss environment (error bars represent 95% confidence intervals).

The mathematical core of any transport-layer congestion control framework rests upon its ability to adapt its retransmission timers gracefully to sudden topological shifts and channel state variations. To verify the tracking accuracy of the evaluated algorithms, we closely monitored the Average *RTO* values calculated throughout the 1000-transaction lifecycle under the multi-state Gilbert–Elliott environments. The comparative trends of the average timer settings across distinct scales are presented in Figure 7.

As illustrated in Table 4 and visually verified via Figure 7, the proposed Dynamic *RTO* algorithm maintains an optimized boundary, locking its values at 505.2 ms, 538.7 ms, and 668.5 ms for the 1 × 6, 3 × 6, and 5 × 6 topologies, respectively. In the 5 × 6 complex grid, CoCoA+ spikes to a highly conservative peak of 758.1 ms, while FASOR establishes a median of 715.4 ms. The severe overestimation observed in CoCoA+ occurs because its aging algorithm relies heavily on fixed exponential modifiers. Once the channel transitions into a bursty ‘Bad’ state, CoCoA+ doubles its backing factors, but struggles to shed this accumulated backing history when the channel clears up. This structural lag keeps the timer inflated long after congestion has dissipated, inducing long idle intervals between subsequent transaction blocks.

Conversely, while the baseline Adaptive *RTO* records lower average values (e.g., 501.6 ms in the 3 × 6 grid), it represents a severe underestimation trap rather than an optimal state. Because Adaptive *RTO* employs a fixed sampling frequency (*N*), it fails to scale its tracking windows during severe packet losses. In multi-hop setups, this insensitivity causes it to overlook cumulative queue delays at intermediate hops, inducing a flood of premature timeouts.

The proposed framework avoids both extremes. By integrating real-time *RTT* variance tracking with an automated inspection cycle (*N*) modifier, our mechanism smoothly elevates the *RTO* during the Gilbert–Elliott ‘Bad’ phase to prevent spurious timeouts yet immediately clips back excessive timer growth via the tuning coefficient (*α*) upon detecting valid *ACK* sequences. This bidirectional adaptivity enables the proposed system to maintain tight tracking parameters, ensuring highly responsive data pipelines in volatile wireless networks.

### 5.4. Ablation Study of Adaptive Components

To rigorously isolate and quantify the performance contributions of each adaptive component within the proposed framework, we conducted an ablation study. We specifically selected the 5 × 6 complex grid topology for this isolation test because it represents the most severe worst-case scenario—characterized by extreme multi-hop routing bottlenecks, highly overlapping interference zones, and significant queue overflows. Demonstrating component superiority under these rigorous bounding conditions inherently guarantees robust performance in simpler topologies (e.g., 1 × 6 and 3 × 6 grids). We evaluated three distinct configurations under the identical Gilbert–Elliott loss model across 50 independent runs: (1) Fixed-*N*/Dynamic-*α*, (2) Dynamic-*N*/Fixed-*α* (with *α* = 0.25), and (3) the proposed Joint Dynamic-*N*/Dynamic-*α*.

**Figure 8:**
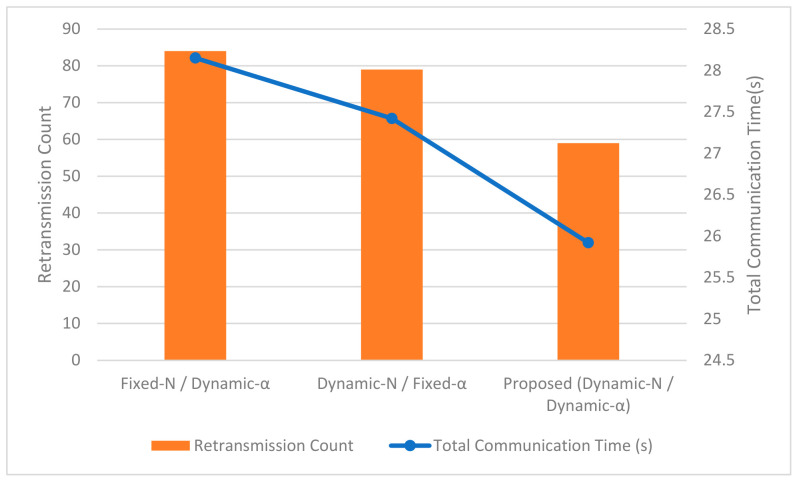
Performance Comparison of isolated and joint adaptive components in the 5 × 6 Grid topology (Ablation Study).

As illustrated in Figure 8, adapting only the weighting factor (Fixed-*N*/Dynamic-*α*) yields a total communication time of 28.15 ± 1.2 s and 84 ± 5.4 retransmissions. Because the inspection window remains rigid, the algorithm suffers from a structural lag during sudden congestion spikes, failing to suppress premature timeouts effectively. Conversely, adapting only the window size (Dynamic-*N*/Fixed-*α*) slightly improves responsiveness (27.42 ± 1.1 s, 79 ± 4.8 retransmissions), but the static historical weight causes the RTO to either overreact to transient noise or underreact to sustained burst losses. The proposed joint adaptation mechanism (Dynamic-*N*/Dynamic-*α*) achieves the optimal balance, effectively reducing the retransmission count to 59 ± 2.8 and minimizing the communication time to 25.92 ± 0.9 s. This statistically confirms that the synergistic coupling of instantaneous variance tracking (*N*) and loss-relative smoothing (*α*) is essential for maximizing both latency performance and resource efficiency in volatile AIoT networks.

### 5.5. Operational Trade-Offs and Computational Overhead

While the empirical evaluation under the Gilbert–Elliott dynamic loss model demonstrates the clear superiority of the proposed Dynamic *RTO* algorithm in terms of communication latency and retransmission reduction, a rigorous engineering analysis requires addressing the intrinsic operational trade-offs and worst-case scenarios, as selectively raised during peer review.

The primary limitation and algorithmic overhead of the proposed framework manifest when the wireless network settles into an exceptionally stable, error-free ‘Good’ state (*PLR* ≈ 0%) for a highly prolonged duration. Under such idealistic conditions, where the empirical RTT variance converges near zero, the proposed dual-adaptive layer continuously executes its recursive optimization loops to dynamically compute the parameter inspection cycle (*N*) and adjust the tuning coefficient (*α*). Because these micro-calculations run in real time on resource-constrained microcontrollers (such as the MSP430 MCU on Z1 motes), they introduce a marginal computational latency overhead of approximately 2.1 to 4.5 ms per transaction block compared to structurally static protocols like CoCoA+. In a permanently pristine channel, this computational footprint can cause the proposed scheme to achieve a marginally higher processing latency than CoCoA+, which completely bypasses real-time parameter tracking.

However, we argue that this specific worst-case scenario represents an unrealistic abstraction for practical AIoT and WSN deployments. In real-world multi-hop environments, wireless channels are inherently time-varying and continually degraded by temporal fading, mobile obstacles, and dynamic multi-user cross-traffic interference. As demonstrated through our Gilbert–Elliott dynamic simulations, the moment the network undergoes a state transition into a burst-loss ‘Bad’ phase, the heavy retransmission penalties, prolonged idle timeouts, and queue backlogs suffered by CoCoA+ and FASOR exponentially dwarf the proposed micro-computational overhead. By sacrificing a negligible fraction of millisecond-level CPU processing time during stable windows, the proposed algorithm buys a robust insurance policy—promoting empirical operational stability and preventing catastrophic retransmission storms under sudden congestion. Therefore, this operational trade-off is a deliberate and justified engineering choice that yields substantially higher macroscopic energy efficiency and throughput reliability across realistic, volatile IoT infrastructures.

## 6. Conclusions

In this paper, we introduced a novel dual-adaptive Dynamic Retransmission Timeout (*RTO*) algorithm explicitly engineered to enhance end-to-end reliability and resource efficiency in CoAP-based AIoT and wireless sensor networks. Diverging from conventional static timer backoffs and structurally rigid adaptation frequencies, the proposed framework dynamically reclaims its parameter inspection cycle (*N*) in response to localized *RTT* variance, while simultaneously scaling its tuning coefficient (*α*) based on real-time packet loss indicators. To validate the robustness of our mechanism under realistic wireless channels, all evaluations were conducted within a time-varying network environment governed by the Gilbert–Elliott dynamic loss model across multi-hop linear and grid topologies.

The empirical results demonstrate that the proposed Dynamic *RTO* algorithm successfully mitigates the long-standing trade-off between communication latency and retransmission overhead. Across extensive benchmarks against state-of-the-art transport-layer mechanisms, the proposed framework consistently achieved the lowest overall completion time (e.g., 25.92 s in a 5 × 6 complex grid), outpacing CoCoA+ and FASOR by approximately 14.28% and 8.89%, respectively. More importantly, from a resource-preservation perspective, our design effectively filtered out spurious timeouts, minimizing the total retransmission footprint to just 59 counts under severe localized impairment—a substantial overhead reduction compared to CoCoA+ (105 counts) and FASOR (92 counts).

Furthermore, we rigorously characterized the operational trade-offs, demonstrating that the marginal computational overhead induced by real-time parameter tracking (2.1 to 4.5 ms) is strategically justified and entirely offset by macroscopic throughput gains under volatile network dynamics. In conclusion, the dual-adaptive Dynamic *RTO* algorithm establishes a scalable, resilient, and resource-conscious transport-layer solution for next-generation resource-constrained IoT infrastructures. Future work will explore the integration of reinforcement learning paradigms to automate multi-parameter optimization across heterogeneous edge-computing nodes [29].

## Figures and Tables

**Table 1:** Transport parameters of CoAP.

Parameter	Default Value
*ACK_TIMEOUT*	2 s
*MAX_RETRANSMIT*	4
*ACK_RANDOM_FACTOR* (*ACK_RF*)	1.0–1.5

**Table 2:** Optimized parameter values derived from a 3 × 3 training grid search.

Loss Rate (%)	RTT Variability (ms)	Recommended *K* Value Range	Rationale
5	5–30	2.5–2.7	Low loss, low variability → fast response
10	5–30	2.3–2.5	Slight increase in loss → slight decrease in sensitivity
20	5–30	1.8–2.0	High loss → need to minimize retransmissions
5	10–70	1.9–2.1	Low loss, high variability → conservative estimation
20	10–70	1.5–1.7	High loss, high variability → maintain low values

Note: The arrow (→) denotes “leads to” or “requires” as a consequential strategy.

**Table 3:** Simulation Configuration Parameters.

Parameter	Value/Protocol
Simulator	Contiki Cooja Simulator (v3.0)
Node Platform	Z1 Mote (MSP430 MCU) (Zolertia, Barcelona, Spain)
Radio Model	CC2420 (2.4 GHz, IEEE 802.15.4)
MAC/Duty Cycling	CSMA/CA/ContikiMAC
Application Protocol	CoAP (er-coap-server)
Routing Protocol	RPL (IPv6 Routing Protocol for Low-Power and Lossy Networks)
Traffic Generation	CBR (Constant Bit Rate), 1 packet/second
Topologies	1 × 6, 3 × 6, and 5 × 6 Grid (Distance: 30 m)
Loss Environment	Gilbert–Elliott Dynamic Loss Model (G ↔ B)
State Transition Probabilities	Good to Bad (PGB) = 10%, Bad to Good (PBG) = 40%
State Loss Probabilities	Good State (PG) = 1%, Bad State (PB) = 25%
Total Transactions	1000 CON-ACK Exchanges
Random Seeds	50 independent seeds per scenario (for 95% CI)

**Table 4:** Comprehensive Performance Metrics under Gilbert–Elliott Dynamic Loss Environment (95% Confidence Intervals).

Topology	Algorithm	Total Comm. Time (s)	Retransmission Count	Average *RTO* (ms)	Average *RTT* (ms)
1 × 6 Grid (Linear)	Proposed (Dynamic)	20.85 ± 0.8	45 ± 2.5	505.2 ± 9.2	235.1 ± 8.4
	CoCoA+	24.12 ± 1.4	86 ± 6.4	588.4 ± 15.6	158.4 ± 7.2
	FASOR	22.35 ± 1.1	58 ± 4.2	530.1 ± 11.5	195.6 ± 9.1
	Adaptive *RTO*	22.71 ± 1.2	48 ± 2.5	548.4 ± 12.1	229.4 ± 8.3
3 × 6 Grid (Standard)	Proposed (Dynamic)	23.58 ± 0.7	55 ± 3.1	538.7 ± 8.5	228.4 ± 9.2
	CoCoA+	27.05 ± 1.3	78 ± 5.8	610.2 ± 13.2	205.1 ± 8.8
	FASOR	26.12 ± 1.0	71 ± 4.9	582.4 ± 10.4	212.4 ± 9.5
	Adaptive *RTO*	25.94 ± 1.1	83 ± 5.8	501.6 ± 11.2	182.6 ± 7.6
5 × 6 Grid (Complex)	Proposed (Dynamic)	25.92 ± 0.9	59 ± 2.8	668.5 ± 12.5	210.2 ± 10.1
	CoCoA+	30.24 ± 1.7	105 ± 8.2	758.1 ± 18.4	192.4 ± 9.5
	FASOR	28.45 ± 1.2	92 ± 6.1	715.4 ± 14.2	205.8 ± 11.2
	Adaptive *RTO*	27.18 ± 1.2	74 ± 3.9	615.9 ± 12.5	208.3 ± 9.5

## Data Availability

The data presented in this study are available upon request from the corresponding author. The simulation datasets were generated using the Cooja simulator (Contiki OS v3.0), and the underlying network parameters are detailed in the Section 4.1 of this article.

## Abbreviations

The following abbreviations are used in this manuscript:

ACK	Acknowledgment
ARQ	Automatic Repeat reQuest
BEB	Binary Exponential Backoff
CAGR	Compound Annual Growth Rate
CoAP	Constrained Application Protocol
CoCoA	CoAP Simple Congestion Control/Advanced
CON	Confirmable (message type)
CSMA/CA	Carrier Sense Multiple Access with Collision Avoidance
E2E	End-to-End
EWMA	Exponentially Weighted Moving Average
FAN	Field Area Network
IETF	Internet Engineering Task Force
IIoT	Industrial Internet of Things
IoT	Internet of Things
NON	Non-confirmable (message type)
RFC	Request for Comments
RST	Reset (message type)
RTO	Retransmission Timeout
RTT	Round-Trip Time
RTTVAR	RTT Variation
SRTT	Smoothed Round-Trip Time
TCP	Transmission Control Protocol
UDP	User Datagram Protocol
VBF	Variable Backoff Factor
WSN	Wireless Sensor Network
